# Impact of pneumococcal urinary antigen testing on clinical outcomes in patients hospitalized with community-acquired pneumonia

**DOI:** 10.1007/s10096-025-05226-1

**Published:** 2025-07-26

**Authors:** Marita Kern, Markus Fally, Lilian Kolte, Pernille Ravn, Thomas Benfield, Simone Bastrup Israelsen

**Affiliations:** 1 Center of Research and Disruption of Infectious Diseases, Department of Infectious Diseases, Copenhagen University Hospital - Amager and Hvidovre, Hvidovre, Denmark; 2 Section for Respiratory Medicine, Department of Internal Medicine, Zealand University Hospital - Roskilde, Roskilde, Denmark; 3 Department of Respiratory Medicine and Infectious Diseases, Copenhagen University Hospital - Nordsjaelland, Hilleroed, Denmark; 4 Department of Internal Medicine, Section for Infectious Diseases, Copenhagen University Hospital - Herlev and Gentofte, Hellerup, Denmark; 5 Department of Clinical Medicine, Faculty of Health and Medical Sciences, Copenhagen University, Copenhagen, Denmark

**Keywords:** Community-acquired pneumonia, Urinary antigen test, *Streptococcus pneumoniae*, Antimicrobial stewardship

## Abstract

To establish a microbiological etiology in severe community-acquired pneumonia (CAP), most guidelines recommend adding urinary antigen tests (UAT) to respiratory sampling. However, their usage and impact remain unclear. We examined the clinical impact of UAT in hospitalized CAP patients. This multicenter cohort study included patients admitted with CAP at three hospitals in Denmark from 2017 to 2020. Logistic regression and propensity-score matching were applied to assess the effect of UAT on 30-day mortality, targeted antibiotic treatment and atypical antibiotic coverage. Propensity-score matching was applied to adjust for confounding. Of 2,449 patients included, 654 (27%) had UAT performed within 48 hours of admission. *Streptococcus pneumoniae* was detected in 8.0% (52/654). 30-day mortality was 11.8% in the tested group and 10.2% in the untested group (adjusted odds ratio [aOR] 1.17, 95% CI 0.83–1.65). Broad-spectrum antibiotics were administered to 47.9% of tested and 42.4% of untested patients (aOR 1.25, 95% CI 1.00-1.55) at discharge. Overall, there were 212 pneumococcal cases, with 30/212 (14%) solely detected by UAT. Of patients with positive pneumococcal UAT, 26% received broad-spectrum antibiotics at discharge, compared with 46% of UAT-negative patients (aOR 0.41, 95% CI 0.18–0.96). Mortality was similar in tested and untested patients. Tested patients received more broad-spectrum antibiotic treatment than untested patients, while patients with positive pneumococcal UAT received more narrow-spectrum antibiotics. Of all pneumococcal cases, 14% were diagnosed exclusively by UAT, suggesting usefulness in antimicrobial stewardship.

**Supplementary Information:**

The online version contains supplementary material available at 10.1007/s10096-025-05226-1.

## Introduction

Community-acquired pneumonia (CAP) is a common disease, with reported annual incidences in Europe ranging from 1.6 to 10.8 cases per 1,000 adults [1]. CAP often affects the elderly and frail and is among the leading infectious causes of death worldwide [2]. 

*Streptococcus pneumoniae* is one of the most common bacterial pathogens causing CAP, accounting for up to 41% of microbiologically verified cases in recent studies [3–6]. *Legionella pneumophila* is less common but associated with severe disease [7]. 

Due to the similarity of symptoms caused by different respiratory pathogens, the clinical presentation of pneumonia is unsuitable for predicting a bacterial etiology [8]. Moreover, several studies have shown that a pathogen is detected in less than half of all cases where microbiological testing is performed [3, 6, 9]. This often results in treatment with broad-spectrum antibiotics, despite consensus that targeted antibiotic treatment is preferable as it is associated with fewer side effects and reduces the occurrence of antibiotic-resistant bacteria [10]. 

Previous studies have suggested that, when combined with blood culture and respiratory samples, urinary antigen tests (UAT) can increase the diagnostic yield, making them a potential tool for guiding antibiotic therapy [3, 11, 12]. Moreover, UAT have turnaround times of less than 24 hours, which is shorter than traditional culture-based methods and could enable earlier antibiotic de-escalation. Several studies have found that patients with a positive pneumococcal UAT were less likely to receive broad-spectrum antibiotics, including piperacillin/tazobactam, cephalosporines, amoxicillin/clavulanic acid, vancomycin, and agents covering for atypical bacteria (*Mycoplasma pneumoniae*,* Chlamydia pneumoniae*,* Legionella pneumophila*), compared with UAT-negative and untested patients [12–15]. Following antibiotic de-escalation, patients with a positive UAT had clinical outcomes comparable to those who continued with one of these broad-spectrum agents [12–14]. However, the low UAT positivity rate in these studies raises doubts about their overall clinical utility [12–14, 16, 17]. Despite this, the number of UAT-positive patients eligible for antibiotic de-escalation significantly exceeded the number of patients who actually underwent antibiotic de-escalation, indicating room for improvement in current prescribing practices [14, 16, 17]. 

Most European guidelines recommend UAT for moderate-severe pneumonia, but their usage in clinical practice and impact on patient outcomes remain uncertain [18–21]. 

Therefore, this study aimed to examine the usage of UAT in hospitalized CAP patients and assess their impact on mortality and antibiotic de-escalation within a Danish hospital setting.

## Methods

### Study design and population

This was a multicentre cohort study of immunocompetent adult patients admitted with CAP at three academic hospitals in Denmark between 1 November 2017 and 30 July 2020. The cohort is an extension of the Optimizing Treatment of Community-Acquired Pneumonia (optiCAP) cohort established by Fally et al. [22].

CAP was defined as the presence of a new infiltrate on the chest X-ray and at least one of the following signs and symptoms: cough, sputum production, dyspnea, core body temperature > 38.0˚C, and auscultatory findings of rales [22]. Patients were excluded if they had been admitted to hospital during the last 14 days, had *Mycobacterium tuberculosis* disease or were immunosuppressed, as previously described [22]. For this study, patients were also excluded if they had not received antibiotic treatment within 24 hours of hospital admission.

### Covariates

Covariates included in the study were age, sex, smoking, number of previous hospitalizations, hospitalization in a specialized ward, and comorbidities. Comorbidities included chronic obstructive pulmonary disease (COPD), congestive heart failure, diabetes, cerebrovascular disease, hypertension, ischemic heart disease, and cancer (Supplementary Table S1). A specialized ward was defined as one specializing in pulmonary medicine or infectious diseases.

We collected data on hematology and blood chemistry, initial chest X-ray, microbiological test results, and antibiotic treatment prior to and during hospitalization. Data on microbiological testing included results from UAT, oropharyngeal swabs, sputum cultures and blood cultures. All sputum samples underwent microscopy to assess their quality before being cultured.

### Disease severity

Disease severity was defined according to the pneumonia severity score CURB-65 that includes confusion, uremia (urea > 7 mmol/l), respiratory rate > 30 per minute, systolic blood pressure < 90 mmHg or diastolic blood pressure ≤ 60 mmHg, and age ≥ 65 years, documented at hospital admission [23]. Patients were classified as having mild disease if they had a CURB-65 score of 0–2 and moderate-severe disease if they had a CURB-65 score of 3–5.

Other markers of disease severity were selected based on previous literature and included plasma C-reactive protein (CRP) levels, peripheral oxygen saturation, and the need for oxygen therapy, all documented at hospital admission (Supplementary Table S1) [24, 25]. 

### Exposure and follow-up

Exposure was defined as having a UAT performed within the first 48 hours of hospital admission, regardless of whether patients were tested before or after initiating antibiotic treatment. The test used in our study was the Immuview^®^ urinary antigen test, which detects all serotypes of *S. pneumoniae* as well as serogroup 1 of *L. pneumophila* in a single sample.

Individuals were followed from day 2 of hospitalization for up to 30 days. To prevent immortal time bias, follow-up began on day 2, ensuring patients were not followed before their exposure status was determined.

### Antibiotic treatment

Broad-spectrum antibiotic treatment was defined as having received therapy with piperacillin/tazobactam, any cephalosporin, any carbapenem or amoxicillin/clavulanic acid (Supplementary Table S2). Atypical antibiotic coverage was defined as treatment targeting *M. pneumoniae*,* L. pneumophila and C. pneumoniae* and included any macrolide or respiratory fluoroquinolone, as specified in Supplementary Table S2.

### Outcome measures

The primary outcome was 30-day mortality. Secondary outcomes included broad-spectrum antibiotic treatment and atypical antibiotic coverage on day three of hospitalization and at discharge. Day three was chosen to reflect the typical timing for the evaluation of microbiological results, based on time of initial sample collection and usual turnaround times. Antibiotic treatment at discharge was included to account for patients discharged before day three. For the secondary outcomes, we exclusively looked at the most recently administered antibiotics. In our analysis of atypical coverage, we excluded any patients testing positive for *L. pneumophila* on UAT, as these patients are more likely to have received atypical coverage than the remaining cohort.

In addition, we compared primary and secondary outcomes in patients with a positive pneumococcal UAT, and UAT-negative patients. Due to the small size of this sub cohort, we only compared antibiotic treatment at discharge, thereby also including patients discharged before day three.

### Data sources

Data on age, sex, smoking, severity scores, microbiological testing, blood works, vital signs, chest x-ray results, number of previous hospitalizations, hospital ward, and antibiotic treatment were retrieved from electronic medical records. Comorbidity data were obtained from the Danish National Patient Registry [26]. 

Data on vital status following hospital stay were obtained from the Danish Civil Registration System [27]. 

### Statistical analyses

We used descriptive statistics to illustrate the distribution of baseline characteristics.

Logistic regression was used to estimate odds ratios (OR) with 95% confidence intervals (CI) for both primary and secondary outcomes. To adjust for potentially confounding baseline covariates, we applied the propensity-score method for all outcomes, to match patients based on their likelihood of having a UAT performed. Patients were matched 1:1 using the nearest neighbor technique with a caliper of 0.2 [28]. Variables for the propensity-score matching were selected based on the literature and included age, sex, plasma CRP levels, peripheral oxygen saturation at admission, any antibiotic treatment prior to hospitalization, smoking status, multilobar infiltration on chest X-ray, CURB-65 score, oxygen treatment at admission, collection of other microbiological tests (blood cultures, sputum cultures and oropharyngeal swabs), number of previous hospitalizations, and certain comorbidities (Supplementary Table S1) [25, 29, 30]. 

For comparing antibiotic treatment in patients with a positive pneumococcal UAT and UAT-negative patients, we also included results from sputum and blood cultures in our propensity score (Supplementary Table S1). The propensity score method was employed for all analyses of primary and secondary outcomes.

Standardized mean differences (SMDs) were used to assess covariate balance between the tested and untested group before and after matching, with a cut-off of 0.1 to deem covariates unevenly distributed, as recommended by guidelines [31–33]. 

Subgroup analyses for the primary and secondary outcomes were conducted, stratifying subjects by age, plasma CRP levels, CURB-65 score, admission to a specialized hospital ward, and extent of infiltration on chest X-ray (Supplementary Table S3). These analyses aimed to explore whether the association between UAT and 30-day mortality and antibiotic treatment varied among patients in different clinical settings and with different levels of disease severity or frailty.

Lastly, we performed sensitivity analyses for all outcomes, where exposure was defined as having a UAT performed within 24 hours instead of 48 hours.

**Fig. 1:**
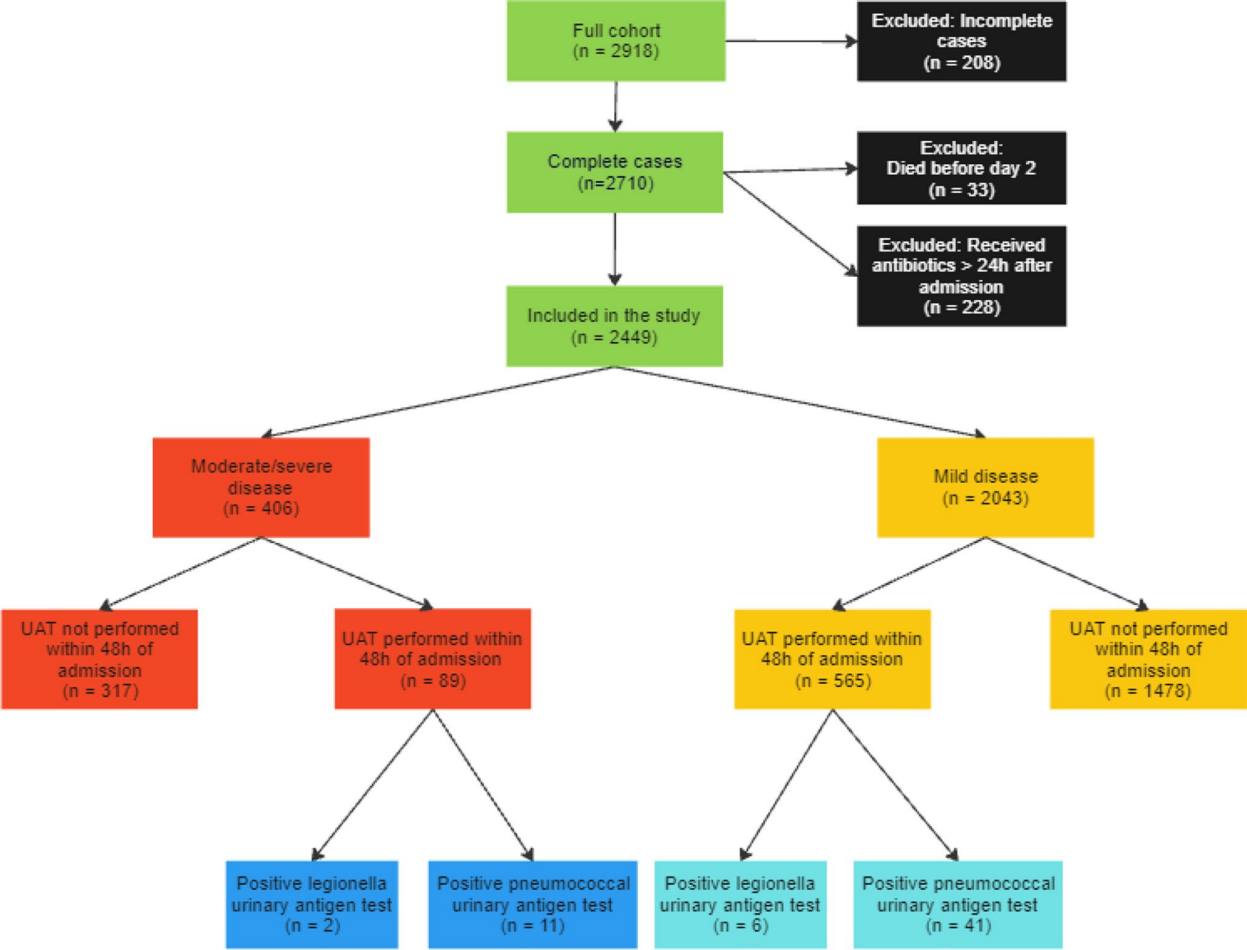
Flowchart of the study population. 208 patients (7%) were excluded due to missing data on antibiotic treatment (1.2%), oxygen saturation (0.03%), oxygen therapy (3.8%), plasma levels of C-reactive protein (1.8%) and CURB-65 -scores (0.7%). Abbreviations: UAT, urinary antigen test. Mild disease: CURB 65-score 0-2. Moderate-severe disease: CURB 65-score 3-5. Created using Miro.com

Since the number of patients with missing data was limited (7%), only complete case analysis was performed. The amount of missing data is shown in Fig. 1.

### Ethical considerations

This study was approved by the Danish Patient Safety Authority (31-1521-101) and the Regional Data Protection Centre (P-2020-1116), with a waiver of informed consent in accordance with Danish legislation.

## Results

### Patient characteristics

During the study period, a total of 2,918 patients hospitalized with CAP were screened. Of these, 2,710 had complete data on all covariates and outcomes. We excluded 228 patients who had not received antibiotic treatment within 24 hours of hospital admission and another 33 patients who died before the commencement of the follow-up period. This resulted in a final cohort of 2,449 patients with CAP, see Fig. 1.

Nearly half of the patients (49.2%) were male, and the median age was 75 years (interquartile range (IQR) 64–84 years). Median length of hospital stay was 4.9 days (IQR 3.0-8.2 days). In the overall cohort, 82.5% presented with mild disease (Table 1).

**Table 1:** Baseline characteristics for patients in the overall cohort stratified according to whether they had UAT performed within 48 hours of hospitalization

	UAT performed within 48 h			
	Overall	No	Yes	SMD
n	2449	1795	654	
Age, median (IQR)	75 (64–84)	76 (66–85)	73 (61–81)	0.22
Male, n (%)	1207 (49.3)	901 (50.2)	306 (46.8)	0.07
Smoking, n (%)	466 (19.0)	323 (18.0)	143 (21.9)	0.14
Chronic obstructive pulmonary disease, n (%)	716 (29.2)	551 (30.7)	165 (25.2)	0.12
Multilobar infiltration on chest x-ray, n (%)	738 (30.1)	552 (30.8)	186 (28.4)	0.05
Diabetes, n (%)	419 (17.1)	307 (17.1)	112 (17.1)	0.001
Cancer, n (%)	192 (7.8)	139 (7.7)	53 (8.1)	0.01
Received antibiotics prior to hospitalization, n (%)	533 (21.8)	383 (21.3)	150 (22.9)	0.04
Peripheral oxygen saturation, median (IQR)	95.0 (93.0–97.0)	95.0 (93.0–97.0)	95.0 (92.3–97.0)	0.08
Ischemic heart disease, n (%)	423 (17.3)	330 (18.4)	93 (14.2)	0.11
Cerebrovascular disease, n (%)	421 (17.2)	321 (17.9)	100 (15.3)	0.07
Congestive heart failure, n (%)	306 (12.5)	233 (13.0)	73 (11.2)	0.06
Hypertension, n (%)	1027 (41.9)	762 (42.5)	265 (40.5)	0.04
CURB-65 score 3–5, n (%)	406 (16.6)	317 (17.7)	89 (13.6)	0.11
Plasma CRP, median (IQR)	110.0 (51–196)	99 (47–180)	150 (63–238)	0.36
Oxygen therapy at admission, n (%)	1155 (47.2)	840 (46.8)	315 (48.2)	0.03
Blood culture^a^, n (%)	2142 (87.5)	1545 (86.1)	597 (91.3)	0.16
Respiratory cultures^a^, n (%)	1316 (53.7)	948 (52.8)	368 (56.3)	0.07
Oropharyngeal swabs^a^, n (%)	1121 (45.8)	715 (39.8)	406 (62.1)	0.46
Previous hospitalizations, n (%)				0.22
0	754 (30.8)	509 (28.4)	245 (37.5)	
1	473 (19.3)	341 (19.0)	132 (20.2)	
2	331 (13.5)	254 (14.2)	77 (11.8)	
3+	891 (36.4)	691 (38.5)	200 (30.6)	
Hospitalized in specialized ward^b^, n (%)	1576 (64.4)	1176 (65.5)	400 (61.2)	0.09

Abbreviations: UAT: urinary antigen test; IQR: interquartile range; CURB-65: confusion, uremia, respiratory rate, blood pressure, age >65; SMD: standardized mean difference; CRP: C-reactive protein

a: Performed within 48 hours

b: Ward specializing in pulmonary medicine or infectious diseases

**Fig. 2:**
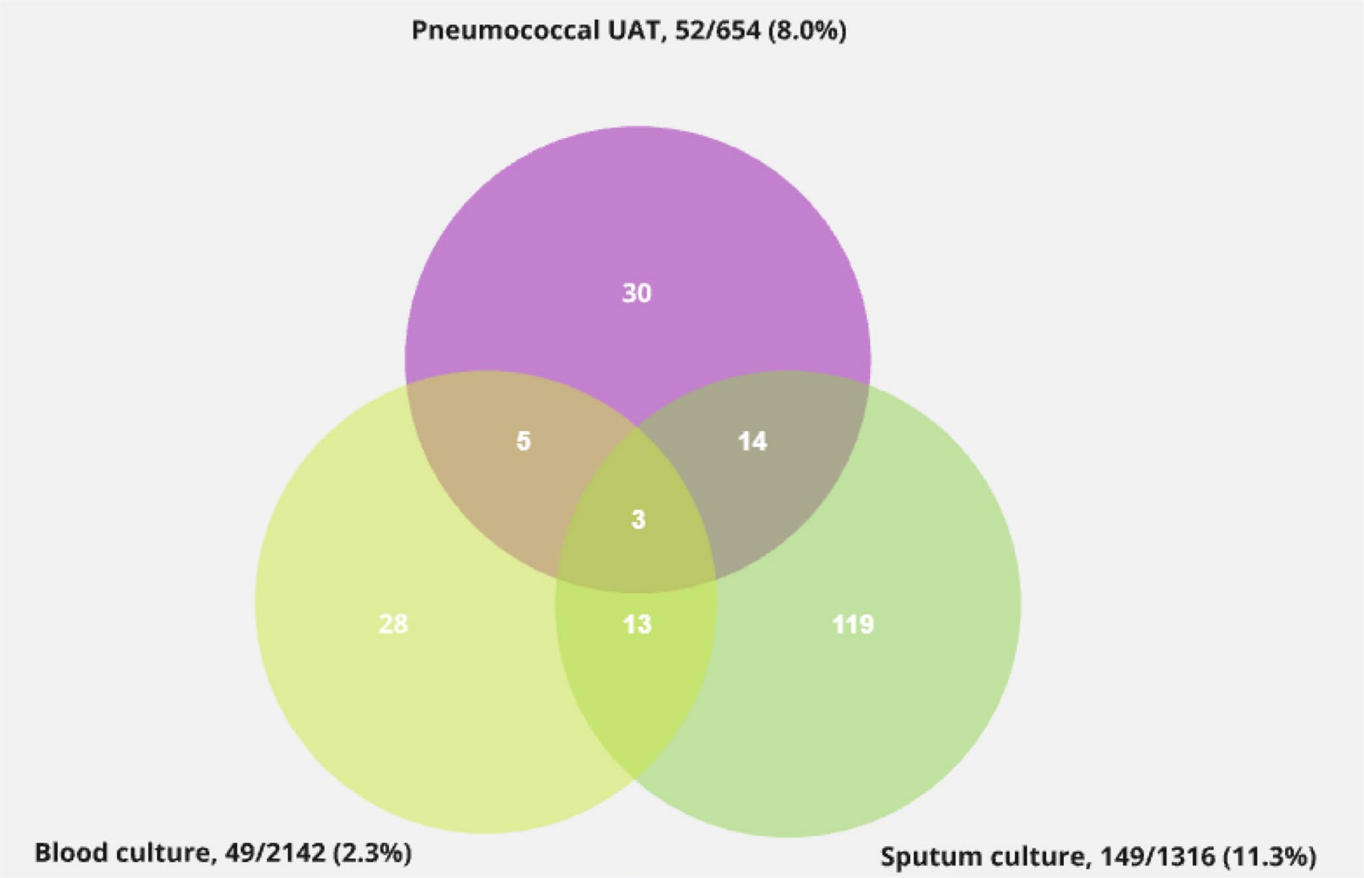
Positivity rates (outside the diagram) and overlaps of urinary antigen tests, sputum cultures and blood cultures performed within 48 hours of hospital admission in the unmatched cohort. Abbreviations: UAT: urinary antigen test. Created using Miro.com

In total, 8.7% (212/2,449) tested positive for *S. pneumoniae* on either a sputum culture, blood culture, or UAT. Among these pneumococcal cases, 52 tested positive on UAT, and 30 cases were exclusively detected by UAT, representing 14.2% (30/212) of all microbiologically verified pneumococcal cases (Fig. 2). Among untested patients in the unmatched cohort, 32/212 (15.1%) pneumococcal cases were detected by blood culture and 107/212 (50.5%) by sputum culture. In the matched control group of untested patients, 48 patients tested positive for *S. pneumoniae* on sputum culture and 20 tested positive on blood culture (Fig. 2). Furthermore, 0.3% (8/2,449) tested positive for *L. pneumophila*, all detected solely by UAT.

Overall, 26.7% (654/2,449) had UAT performed within the first 48 hours of hospitalization. Among these, 8.0% (52/654) tested positive for *S. pneumoniae* antigen and 1.2% (8/654) tested positive for *L. pneumophila* antigen (Fig. 1). Of the 52 patients with a positive pneumococcal UAT, all except two had either a sputum or blood culture performed. Among patients with mild disease, 565/2043 (27.7%) had UAT performed within 48 hours of hospitalization, compared to 89/406 (21.9%) in the group with moderate-severe disease. The UAT positivity rate was 7.3% (41/565) among patients with mild disease and 12.4% (11/89) in the group with moderate-severe disease (Supplementary Table S4).

Patients who had UAT performed were younger, had a higher smoking rate, higher plasma CRP levels, fewer previous hospitalizations, and a higher frequency of performed sputum and blood culture in comparison with patients who did not have UAT performed (Table 1). Disease severity was similar in the tested and untested groups. Of the 654 tests performed, 78% were performed at one of the three hospitals (Supplementary Table S5).

Due to the low number of positive Legionella antigen tests, it was not possible to conduct meaningful analyses of 30-day mortality and antibiotic treatment in patients with positive and negative test results.

### Propensity score matching

All 654 subjects tested with UAT within 48 hours of admission were matched with subjects who were either not tested or tested more than 48 hours after hospital admission (Table 2). This resulted in a matched cohort of 1308 individuals.

**Table 2:** Baseline characteristics for patients in the propensity score matched cohort stratified according to whether they had UAT performed within 48 hours of hospitalization

	UAT performed within 48 h		
	No	Yes	SMD
n	654	654	
Age, median (IQR)	73 (61–82)	73 (61–81)	0.046
Male, n (%)	306 (46.8)	306 (46.8)	< 0.001
Smoking, n (%)	136 (20.8)	143 (21.9)	0.050
Chronic obstructive pulmonary disease, n (%)	162 (24.8)	165 (25.2)	0.011
Multilobar infiltration on chest x-ray, n (%)	191 (29.2)	186 (28.4)	0.017
Diabetes, n (%)	99 (15.1)	112 (17.1)	0.054
Cancer, n (%)	50 (7.6)	53 (8.1)	0.017
Received antibiotics prior to hospitalization, n (%)	155 (23.7)	150 (22.9)	0.018
Peripheral oxygen saturation, median (IQR)	95 (93–96)	95 (92–97)	0.006
Ischemic heart disease, n (%)	92 (14.1)	93 (14.2)	0.004
Cerebrovascular disease, n (%)	112 (17.1)	100 (15.3)	0.050
Congestive heart failure, n (%)	62 (9.5)	73 (11.2)	0.055
Hypertension, n (%)	270 (41.3)	265 (40.5)	0.016
CURB-65-score 3–5, n (%)	96 (14.7)	89 (13.6)	0.031
Plasma CRP, median (IQR)	138 (66–230)	150 (63–238)	0.060
Oxygen therapy at admission, n (%)	296 (45.3)	315 (48.2)	0.058
Blood culture^a^, n (%)	592 (90.5)	596 (91.1)	0.021
Sputum culture^a^, n (%)	369 (56.4)	368 (56.3)	0.003
Oropharyngeal swab^a^, n (%)	405 (61.9)	406 (62.1)	0.003
Previous hospitalizations, n (%)			0.030
0	252 (38.5)	245 (37.5)	
1	125 (19.1)	132 (20.2)	
2	77 (11.8)	77 (11.8)	
3+	200 (30.6)	200 (30.6)	
Hospitalized in a specialized ward^b^, n (%)	434 (66.4)	400 (61.2)	0.108

Abbreviations: UAT: urinary antigen test; IQR: interquartile range; CURB-65: confusion, uremia, respiratory rate, blood pressure, age >65; SMD: standardized mean difference; CRP: C-reactive protein

a: Performed within 48 hours

b: Ward specializing in pulmonary medicine or infectious diseases

After propensity-score matching, all SMDs for covariates included in the matching were less than 0.1 (Table 2, Supplementary Fig. S1).

In a sub cohort, we matched the 50 patients with a positive pneumococcal UAT and available results from either sputum or blood culture, with UAT-negative patients.

### 30-day mortality

**Fig. 3:**
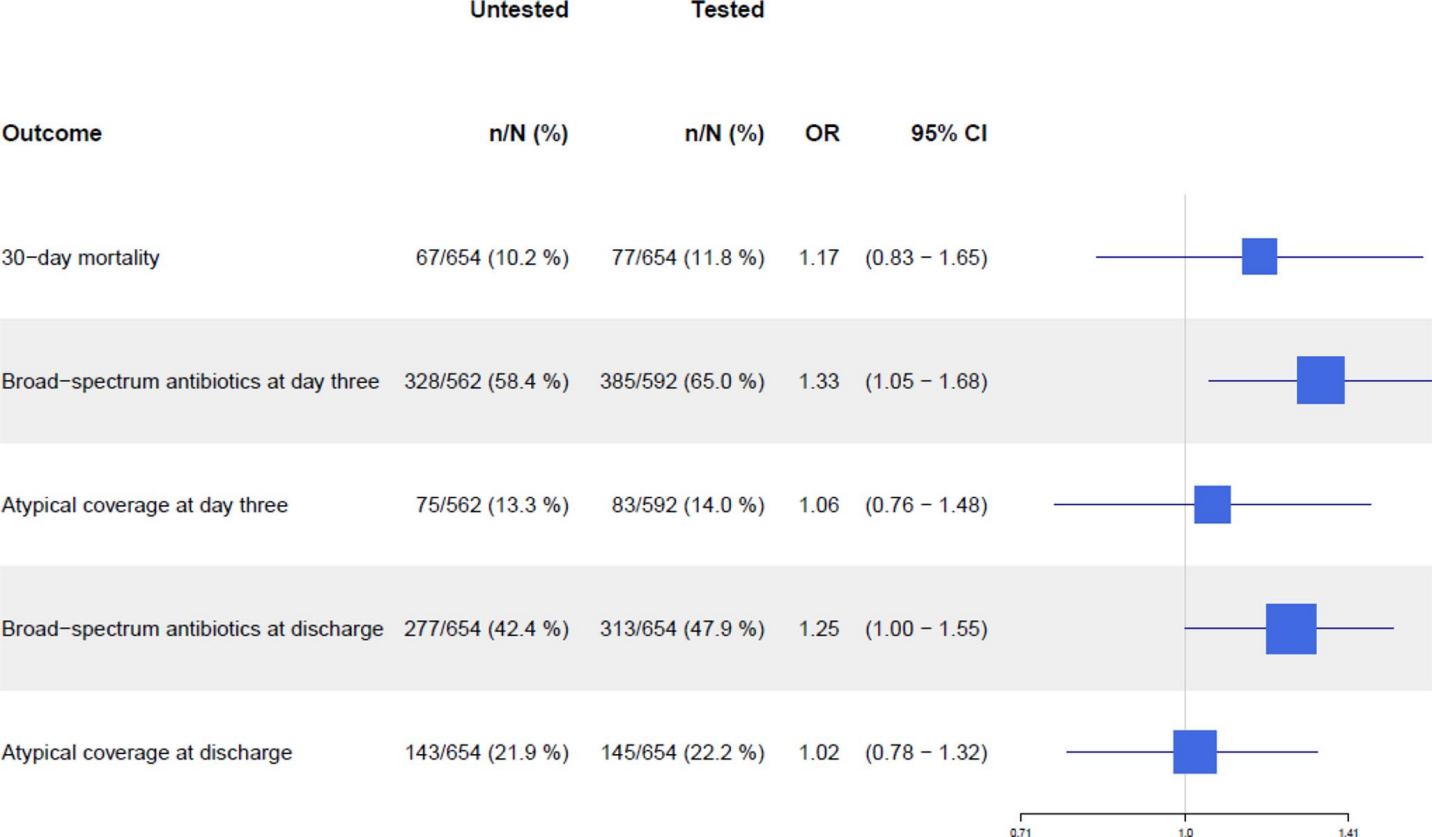
Forest plot of primary and secondary outcomes in propensity score matched tested and untested patients. Abbreviations: OR: Odds ratio; CI: Confidence interval. Data on antibiotic treatment at day three were only available for 1,154 patients in the matched cohort, due to early discharge. Created in R Studio

The UAT-tested group had a 30-day mortality of 11.8% (77/654), compared with 10.2% (67/654) in the untested group (adjusted OR (aOR) 1.17, 95% CI 0.83–1.65) (Fig. 3). Among 52 patients with a positive pneumococcal UAT, the 30-day mortality was 13.5% (7/52), while it was 17.3% (9/52) in patients with a negative UAT (aOR 0.74, 95% CI 0.25–2.17) (Table 3).

**Table 3:** Primary and secondary outcomes in patients with a positive or negative Pneumococcal UAT

	Pneumococcal UAT		
	Positive *n*/*N* (%)	Negative *n*/*N* (%)	Adjusted^a^ odds ratio [95% CI]
30-day mortality	7/52 (13.5)	9/52 (17.3)	0.74 [0.25–2.17]
Treatment with broad-spectrum antibiotics at discharge	13/50 (26.0)	23/50 (46.0)	0.41 [0.18–0.96]
Treatment with atypical antibiotic coverage at discharge	11/50 (22.0)	12/50 (24.0)	0.89 [0.35–2.26]

Abbreviations: UAT: urinary antigen test

a: Adjusted for age, sex, plasma CRP levels, peripheral oxygen saturation at admission, any antibiotic treatment prior to hospitalization, smoking status, multilobar infiltration on chest X-ray, CURB-65 score, oxygen treatment at admission, results from other microbiological testing, number of previous hospitalizations, and comorbidities

### Broad-spectrum antibiotic treatment

At day three, 65.0% (385/592) received broad-spectrum antibiotic treatment in the tested group, compared with 58.4% (328/562) in the untested group (aOR 1.33, 95% CI 1.05–1.68). At discharge, 47.9% (313/654) of patients in the tested group were receiving broad-spectrum antibiotic treatment, compared with 42.4% (277/654) in the untested group (aOR 1.25, 95% CI 1.00–1.55) (Fig. 3).

At discharge, 26.0% (13/50) of patients with a positive pneumococcal UAT were treated with broad-spectrum antibiotics, while 46.0% (23/50) received broad-spectrum antibiotics in the group with a negative test result (aOR 0.41, 95% CI 0.18–0.96) (Table 3).

### Atypical antibiotic coverage

At day three, 14.0% (83/592) of the tested group received atypical antibiotic coverage, compared with 13.3% (75/562) in the untested group (aOR 1.06, 95% CI 0.76–1.48). At discharge, 22.2% (145/654) in the tested group had atypical antibiotic coverage, while 21.9% (143/654) had atypical antibiotic coverage in the untested group (aOR 1.02, 95% CI 0.78–1.32) (Fig. 3). Among patients with a positive pneumococcal test, 22.0% of patients (11/50) had atypical antibiotic coverage at discharge, compared with 24.0% (12/50) in UAT-negative patients (aOR 1.73, 95% CI 0.61–4.9) (Table 3).

### Subgroup analyses

In patients with moderate-severe disease, the 30-day mortality was 25.8% (23/89) in the tested group and 34.8% (31/89) in the untested group. In patients with mild disease, the tested group had a 30-day mortality of 9.6% (54/565), compared to 7.1% (40/565) in the untested group (Supplementary Fig. S2).

In patients with moderate-severe disease, 53.9% (48/89) in the tested group received broad-spectrum antibiotic treatment at discharge, compared with 50.6% (45/89) in the untested group (Supplementary Fig. S5). For the remaining results from the subgroup analyses on broad-spectrum antibiotic treatment and atypical antibiotic coverage, see Supplementary Figs. S3, S4 and S6.

### Sensitivity analyses

When looking at patients tested within 24 hours of admission, we found that the association between UAT and 30-day mortality was similar to that observed in patients tested within 48 hours. We also observed comparable associations between UAT and the use of broad-spectrum antibiotics, as well as atypical antibiotic coverage (Supplementary Table S6).

## Discussion

### Main findings

In this cohort study of 2,449 patients hospitalized with CAP, we identified 212 cases of pneumococcal pneumonia, with 14% exclusively detected by UAT. Overall, we found comparable 30-day mortality rates in patients who underwent UAT testing and those who did not. Interestingly, UAT-tested patients were not less likely to receive broad-spectrum antibiotics at day three of hospitalization or at discharge, compared with untested patients. Among UAT-untested patients, 139 pneumococcal cases were identified, 68 of which were in the matched untested group. Patients who tested positive for *S. pneumoniae* on UAT more often received treatment with narrow-spectrum antibiotics at discharge, compared with UAT-negative patients. Finally, UAT testing was not associated with the use of atypical antibiotic coverage.

### Research in context

Our study’s finding of no association between UAT testing and mortality aligns with previous findings of other studies [12, 34]. However, our results should be interpreted with caution due to the relatively low number of events.

The association between a positive pneumococcal UAT and antibiotic de-escalation has been consistently observed in several studies [12–14]. However, similarly to those studies, the low positive rate of UAT in our study meant that only a minority of patients underwent antibiotic de-escalation following a positive test.

Despite the reported low positive rates, several studies have found that UAT contribute substantially to the overall diagnostic yield in patients with CAP, suggesting its utility as a supplement to conventional microbiological tests [3, 12]. In our study, cases of *S. pneumoniae* exclusively diagnosed by UAT accounted for 14% of all microbiologically confirmed pneumococcal pneumonia. This represents a higher additional diagnostic yield compared to recent studies, despite the overall UAT positive rate being lower than rates reported in other studies [3, 11, 12]. 

In our study, the majority of UAT were performed in patients with mild disease, contrary to most guidelines that recommend testing individuals with severe disease only. However, positive rates were higher in individuals with moderate-severe disease, suggesting that disease severity affects the positivity rate and thus supporting the recommendations in current guidelines. The majority of tests were performed at one of the included hospitals, likely due to its high patient volume.

Notably, a quarter of patients with a positive pneumococcal UAT in this study continued to receive broad-spectrum therapy at day three, even though the majority of *S. pneumoniae* isolates in Denmark are susceptible to penicillin. This suggests variability in antibiotic de-escalation practices among clinicians in response to a positive pneumococcal test result, highlighting potential for improvement in clinical prescription and decision practices. Unfortunately, we were unable to ascertain reasons for continuing broad-spectrum therapy, which may include factors such as uncertainty about the test result, polymicrobial infections, penicillin allergies, or other concerns related to narrowing antibiotic therapy.

It is unclear why tested patients received more broad-spectrum antibiotics in this study. However, the number of positive pneumococcal cases was higher in the untested group than in the tested group, which could explain part of this lack of difference. As the tested group had more mild disease it is unlikely that lack of de-escalation was related to disease severity. Nevertheless, it is possible that factors related to disease severity or frailty, beyond those accounted for in our analysis, may have influenced this outcome.

These factors could potentially affect both the likelihood of undergoing UAT-testing and the decision to continue broad-spectrum therapy. For instance, a higher level of frailty is associated with increased risk of clinical relapse and could lead to a reluctance among clinicians to de-escalate antibiotic treatment.

### Strengths and limitations

Our study has several strengths. Firstly, it includes a large study population with granular in-hospital data, including detailed antibiotic treatment, facilitating analysis of multiple endpoints. Secondly, we were able to follow patients after hospital discharge, providing comprehensive insight into post-discharge clinical outcomes. Lastly, our use of propensity score matching with detailed clinical data enabled us to effectively account for numerous potential confounding variables.

This study also has some limitations. First, the limited number of mortality events, the primary outcome in this study, poses challenges in drawing any firm conclusions from our analysis. Second, the low proportion of patients with moderate to severe disease having UAT performed limits our ability to test the impact in this subgroup. In addition, we could not account for possible false positives due to pneumococcal vaccination or nasopharyngeal colonization as these data were unavailable. However, we adjusted for several chronic comorbidities, such as chronic respiratory disease and diabetes mellitus, that qualify patients for subsidized pneumococcal vaccination in Denmark. Lastly, our analysis did not include all markers of frailty and disease severity, possibly suggesting the presence of unadjusted confounders.

## Conclusion

In conclusion, patients hospitalized with CAP who underwent UAT had similar 30-day mortality rates but received broad-spectrum antibiotics more frequently compared to those who did not.

Among patients with positive pneumococcal UAT results, a significantly higher proportion were discharged with narrow-spectrum antibiotics compared to UAT-negative patients Additionally, UAT exclusively identified 14% of all pneumococcal cases, highlighting its potential role in antimicrobial stewardship.

Future studies should focus on evaluating the cost-effectiveness of performing UAT in patients with severe disease, considering the otherwise low positivity rate that limits their clinical utility.

## Electronic supplementary material

Below is the link to the electronic supplementary material.

Supplementary Material 1

## Data Availability

Raw data for this study are not publicly available due to privacy or ethical restrictions.
