# Supplementary material for: Impact of pneumococcal urinary antigen testing on clinical outcomes in patients hospitalized with community-acquired pneumonia
Source: Eur J Clin Microbiol Infect Dis. 2025 Jul 26;44(11):2635–44. doi: 10.1007/s10096-025-05226-1 (PMC12619800; doi:10.1007/s10096-025-05226-1)
Supplement: Supplementary file 1 — Supplementary Material 1 [file 10096_2025_5226_MOESM1_ESM.docx]

Supplementary material

**Supplementary Table S1:** Covariates included in the propensity score model

| Covariate | Data source | Definition | Form | Categories |
| --- | --- | --- | --- | --- |
| Age | Danish Civil Registration System |  | Linear, quadratic | N/A |
| Sex | Danish Civil Registration System |  | Indicator | Female/male |
| Smoking | Electronic medical record | 1 - Current smoker  2 - Former smoker  3 - Never smoker  9 - Not documented | 4 categories | 1, 2, 3, 9 |
| Antibiotics before admission | Electronic medical record |  | Dichotomous | Yes/No |
| Diabetes mellitus^a^ | Danish National Patient Registry; Danish National Prescription Registry | ICD-10: E10, E11, E13, E14;  ATC: A10A, A10B | Indicator | Yes/No |
| Chronic obstructive pulmonary disease^a^ | Danish National Patient Registry | ICD-10: J44 | Indicator | Yes/No |
| Cancer (excl. non-melanoma skin cancer)^a^ | Danish National Patient Registry | ICD-10: C0-C3, C40, C41, C43, C45, C46-C49, C5, C6, C70-C76, C80-C85, C883, C887, C889, C900, C901, C91-C93, C940, C941, C942, C943, C9451, C947, C95, C96 | Indicator | Yes/No |
| Cerebrovascular disease^a^ | Danish National Patient Registry | ICD-10: I60-I69, G45, G46 | Indicator | Yes/No |
| Hypertension^a,b^ | Danish National Patient Registry; Danish National Prescription Registry | ICD-10: I10, I11, I12, I13, I15;  ATC: ≥1 combination tablets (C02L, C02N, C03EA, C07B, C07C, C07D, C07E, C07FB, C07FX01, C08CA55, C08DA51, C08G, C09BA, C09BB, C09BX, C09DA, C09DB, C09DX, C09XA52, C09XA53, C09XA54), or a combination of ≥2 of the following classes (angiotensin converting enzyme inhibitors (C09A), angiotensin II receptor blockers (C09C), renin inhibitors (C09XA01 and C09XA02), calcium channel blockers (C08C except C08CA55, C08D except C08DA51, and C08E), beta blockers (C07A and C07FX except C07FX01), antiadrenergic agents (C02A, C02B, and C02C), non-loop diuretics (C02DA, C03A, C03B, C03D, and C03X), vasodilators (C02DB, C02DD, and C02DG)​ | Indicator | Yes/No |
| Ischemic heart disease^a^ | Danish National Patient Registry | ICD-10: I20, I21, I22, I23, I24, I25 | Indicator | Yes/No |
| Congestive heart failure^a^ | Danish National Patient Registry | ICD-10: I50, I110, I130, I132 | Indicator | Yes/No |
| Health care utilization | Danish National Patient Registry | Number of admissions within past 3 years: 0, 1, 2, 3+ | 4 categories | 0, 1, 2, 3 |
| Disease severity | Electronic medical record | 1 – Mild disease (CURB-65 score 0-2)  2 – Moderate-severe disease (CURB-65 score 3-5) | 2 categories | 1, 2 |
| Peripheral oxygen saturation at admission | Electronic medical record |  | Linear,  quadratic | N/A |
| Oxygen therapy at admission | Electronic medical record |  | Indicator | Yes/No |
| Multilobar infiltrates on chest X-ray | Electronic medical record | 0 - Unilobar  1 - Multilobar | Indicator | 0, 1 |
| C-reactive protein level at admission | Electronic medical record |  | Linear,  quadratic | N/A |
| Blood cultures performed within 48 hours | Electronic medical record |  | Indicator | Yes/No |
| Oropharyngeal swabs taken within 48 hours | Electronic medical record | Test for Respiratory viruses, Chlamydophila pneumoniae, Mycoplasma pneumoniae | Indicator | Yes/No |
| Respiratory tract cultures performed within 48 hours | Electronic medical record |  | Indicator | Yes/No |
| Results from blood cultures or respiratory tract cultures performed within 48 hours^b^ | Electronic medical record | Detection of S. pneumoniae | Indicator | Positive/  Negative |

**Supplementary Table S1:**

Abbreviations: CURB-65: confusion, uremia, respiratory rate, blood pressure, age > 65; CRP: C-reactive protein; ICD-10: International Classification of Diseases, 10^th^ revision; ATC: Anatomical Therapeutic Chemical Classification

a: Look back: 10 years before admission

b: Only included in the model for analysis of patients with a positive vs negative pneumococcal UAT

**Supplementary Table S2:** Definitions of broad-spectrum and atypical antibiotic coverage

| Outcome | Data source | Definition (ATC codes) | Form | Categories |
| --- | --- | --- | --- | --- |
| Broad-spectrum antibiotic therapy | Medical record | Piperacillin/tazobactam (J01CR05), any cephalosporin (J01DD01; J01DC02; J01DD04; J01DD02; J01DB01; J01DD52), any carbapenem J01DH02; J01DH03) or amoxicillin/clavulanic acid (J01CR02) | Indicator | Yes/No |
| Atypical antibiotic coverage | Medical record | Any macrolide (J01FA10; J01FA06; J01FA01; J01FA09) or respiratory fluorquinolone (J01MA02, J01MA14) | Indicator | Yes/No |

**Supplementary Table S3:** Stratifying variables for the subgroup analyses

**Supplementary Table S3:**

Abbreviations: CRP: C-reactive protein (mg/L), CURB-65: confusion, uremia, respiratory rate, blood pressure, age > 65

| Covariate | Data source | Definition | Form | Categories |
| --- | --- | --- | --- | --- |
| Age | Electronic medical record | 1 – < median age (75)  2 – ≥ median age (75) | 2 categories | 1, 2 |
| Infiltration on chest x-ray | Electronic medical record | 0 – Unilobar  1 – Multilobar | 2 categories | 1, 2 |
| C-reactive protein level at admission | Electronic medical record | 1 – < 100 mg/L  2 – ≥ 100 mg/L | 2 categories | 1, 2 |
| Disease severity | Electronic medical record | 1 – Mild disease (CURB-65 score 0-2)  2 – Moderate-severe disease (CURB-65 score 3-5) | 2 categories | 1, 2 |
| Type of hospital ward | Electronic medical record | 1 – specialized ward (infectious diseases ward or pulmonary medicine ward)  2 – non-specialized ward (defined as any other type of ward) | 2 categories | 1, 2 |

**Supplementary Table S4:** UAT positivity rate stratified by disease severity (CURB65-score)

| Etiology | Mild disease^a^  n/N (%) | Moderate-severe disease^b^  n/N (%) |
| --- | --- | --- |
| *S. pneumoniae* | 41/565 (7.3) | 11/89 (12.4) |
| Total^a^ | 47/565 (8.3) | 13/89 (14.6) |

**Supplementary Table S4**

Abbreviations: S. pneumoniae: Streptococcus pneumoniae

a: Including urinary antigen tests positive for *Legionella pneumophila*

a: CURB 65-score 0-2
b: CURB 65-score 3-5

:

**Supplementary Table S5** Number of urinary antigen tests performed within 48 hours of admission by hospital

| Hospital | Number of UAT performed  n/N (%) |
| --- | --- |
| Hospital 1 | 69 (10.6) |
| Hospital 2 | 72 (11.0) |
| Hospital 3 | 513 (78.4) |

**Supplementary Table S5:**

Abbreviations: UAT: urinary antigen test

**Supplementary Table S6:** Sensitivity analysis of overall outcomes with exposure redefined as having UAT performed within 24 hours of hospitalization

**UAT performed within 24 hours**

|  | Yes  n/N (%) | No  n/N (%) | Adjusted^a^ odds ratio [95 % CI] |
| --- | --- | --- | --- |
| 30-day mortality | 67/585 (11.5) | 66/585 (11.3) | 1.02 [0.71 - 1.46] |
| Day three  Treatment with broad-  spectrum antibiotics | 347/524 (66.2) | 310/502 (61.8) | 1.21 [0.94 – 1.57] |
| Atypical antibiotic coverage | 69/524 (13.2) | 69/502 (13.7) | 0.95 [0.66 – 1.36] |
| Discharge  Treatment with broad-  spectrum antibiotics | 284/585 (48.5) | 256/585 (43.8) | 1.21 [0.96 – 1.53] |
| Atypical antibiotic coverage | 128/585 (21.9) | 131/585 (22.4) | 0.97 [0.74 – 1.28] |

**Supplementary Table S6:**

Abbreviations: UAT: urinary antigen test

a: Adjusted for age, sex, plasma CRP levels, peripheral oxygen saturation at admission, any antibiotic treatment prior to hospitalization, smoking status, multilobar infiltration on chest X-ray, CURB-65 score, oxygen treatment at admission, results from microbiological testing, number of previous hospitalizations, and comorbidities

**Supplementary Figure 1:** Plot of standardized mean differences


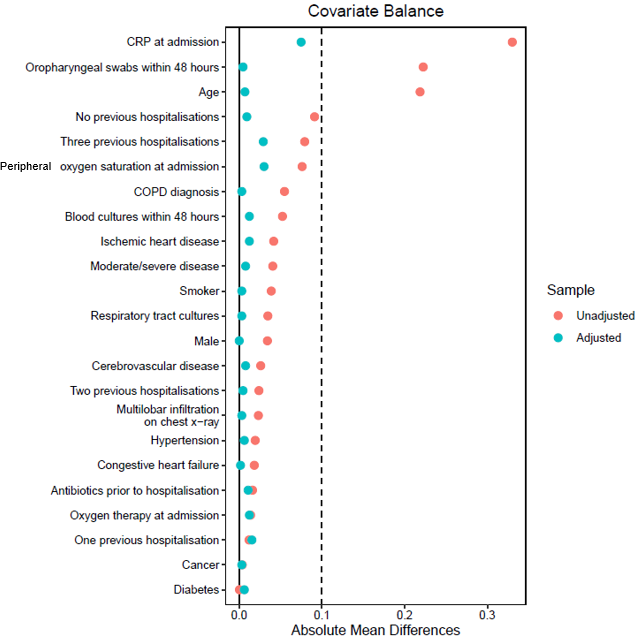


**Supplementary Fig. 1** Plot of standardized mean differences in confounding variables between tested and untested patients before and after propensity score matching

Unadjusted: Before propensity score matching

Adjusted: After propensity score matching

Abbreviations: COPD: Chronic obstructive pulmonary disease; CRP: C-reactive protein (mg/L)

Created in R Studio.

**Supplementary Figure 2:** Forest plot of subgroup analyses for 30-day mortality in tested and untested patients


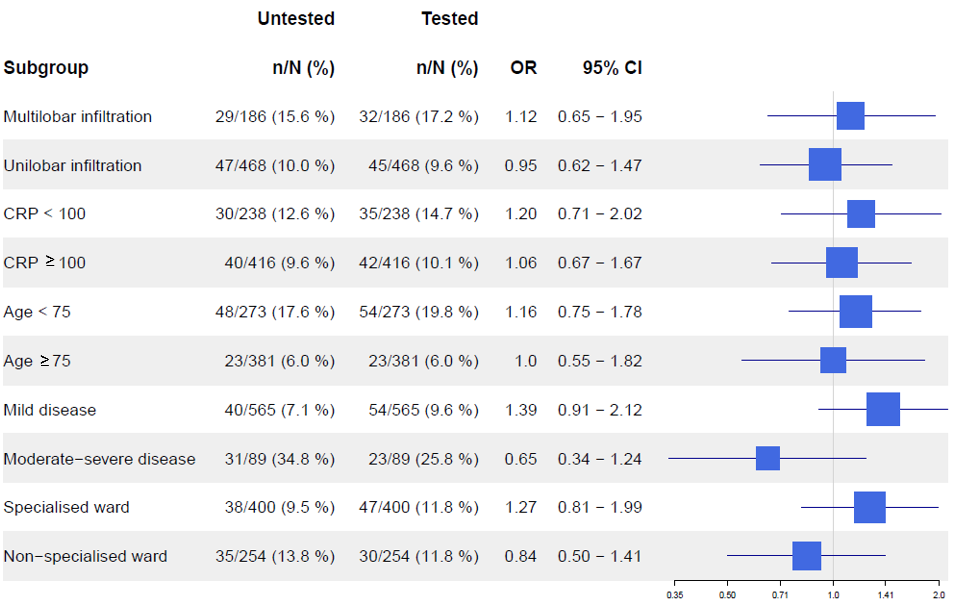


**Supplementary Figure 3:** Forest plot of subgroup analyses for broad-spectrum antibiotic treatment in tested and untested patients at day three

**Supplementary Fig. 2**

Abbreviations: CRP: C-reactive protein (mg/L)

Mild disease: CURB-65 score 0-2
Moderate-severe disease: CURB-65 score 3-5
Specialized ward: Ward specializing in pulmonary medicine or infectious diseases.

Created in R Studio.


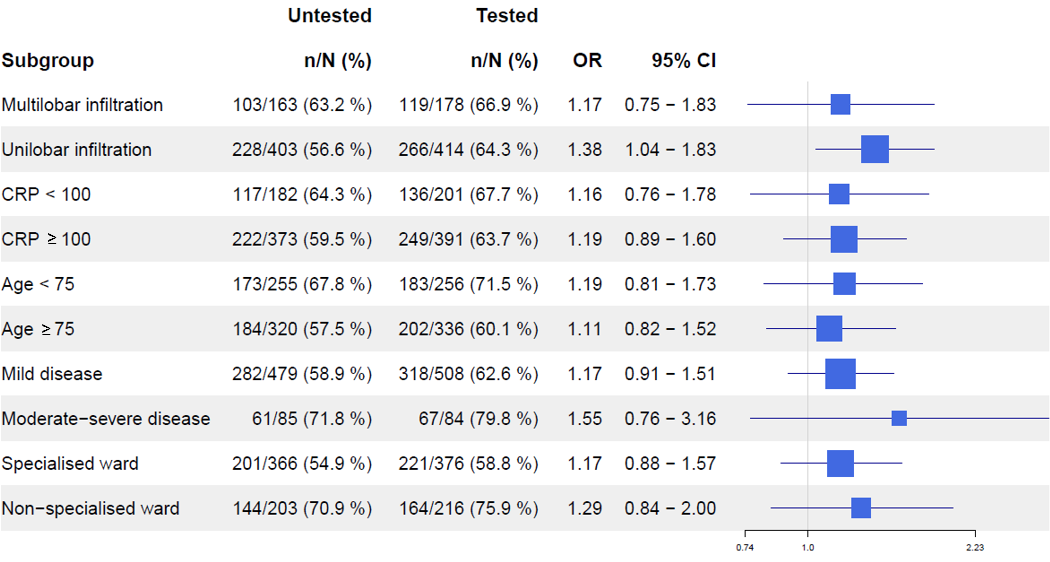


**Supplementary Fig. 3**

Abbreviations: CRP: C-reactive protein (mg/L)

Mild disease: CURB-65 score 0-2
Moderate-severe disease: CURB-65 score 3-5
Specialized ward: Ward specializing in pulmonary medicine or infectious diseases.

Created in R Studio.

**Supplementary Figure 4:** Forest plot of subgroup analyses for atypical antibiotic coverage in tested and untested patients at day three


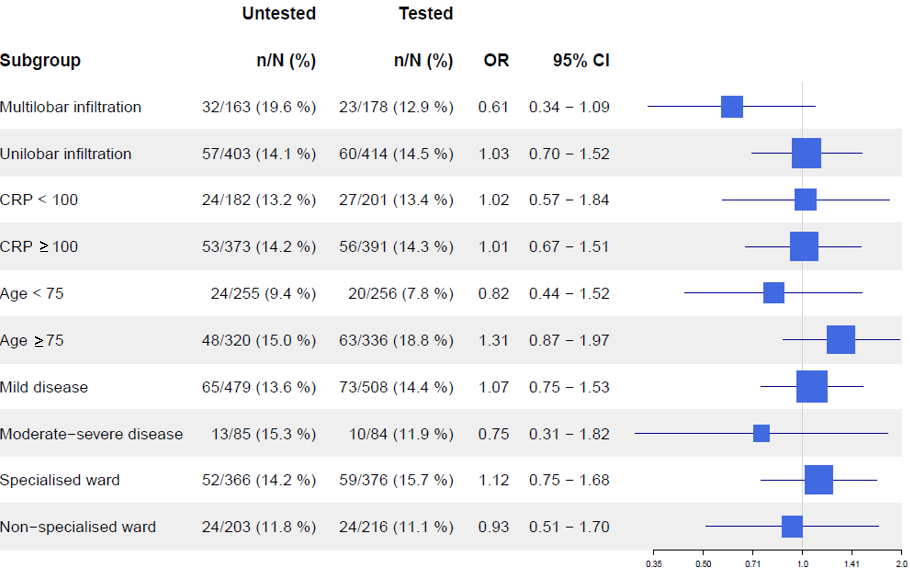


**Supplementary Fig. 4**

Abbreviations: CRP: C-reactive protein (mg/L)

Mild disease: CURB-65 score 0-2
Moderate-severe disease: CURB-65 score 3-5
Specialized ward: Ward specializing in pulmonary medicine or infectious diseases.

Created in R Studio.

**Supplementary Figure 5:** Forest plot of subgroup analyses for broad-spectrum antibiotic treatment in tested and untested patients at discharge


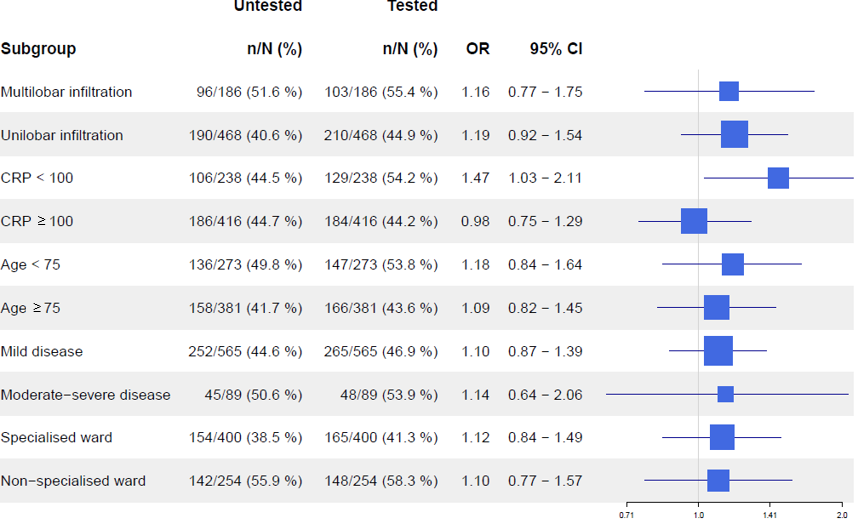


**Supplementary Fig. 5**

Abbreviations: CRP: C-reactive protein (mg/L)

Mild disease: CURB-65 score 0-2
Moderate-severe disease: CURB-65 score 3-5
Specialized ward: Ward specializing in pulmonary medicine or infectious diseases.

Created in R Studio.

**Supplementary Figure 6:** Forest plot of subgroup analyses for atypical antibiotic coverage in tested and untested patients at discharge


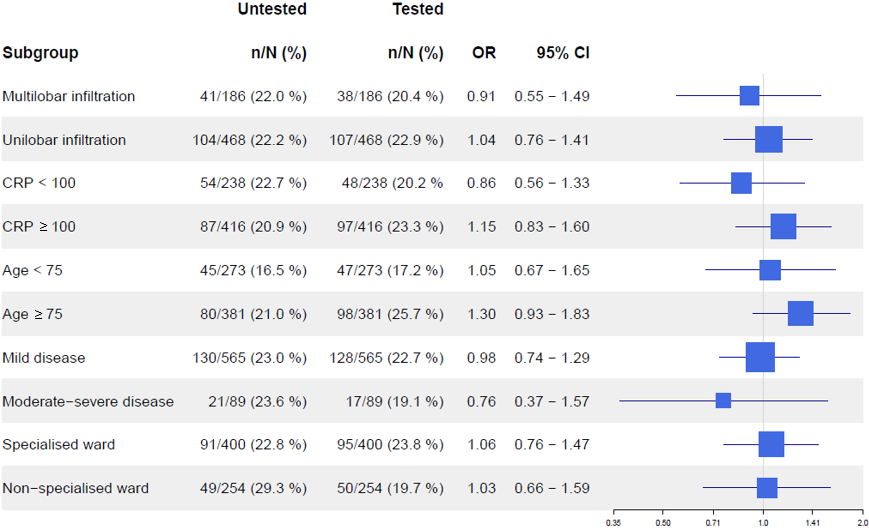


**Supplementary Fig. 6**

Abbreviations: CRP: C-reactive protein (mg/L)

Mild disease: CURB-65 score 0-2
Moderate-severe disease: CURB-65 score 3-5
Specialized ward: Ward specializing in pulmonary medicine or infectious diseases.

Created in R Studio.
